# Disrupted Effective Connectivity within the Fronto-Thalamic Circuit in Pontine Infarction: A Spectral Dynamic Causal Modeling Study

**DOI:** 10.3390/brainsci14010045

**Published:** 2024-01-01

**Authors:** Huiyou Chen, Qianqian Mao, Yujie Zhang, Mengye Shi, Wen Geng, Yuehu Ma, Yuchen Chen, Xindao Yin

**Affiliations:** Department of Radiology, Nanjing First Hospital, Nanjing Medical University, Nanjing 210006, China; chenchenxiaoyou@163.com (H.C.); njmumqq@163.com (Q.M.); zhangyujie3666@163.com (Y.Z.); shimengye1988@126.com (M.S.); sdygw1636@163.com (W.G.); xddcnn@163.com (Y.M.); chenyuchen1989@126.com (Y.C.)

**Keywords:** pontine stroke, resting-state functional magnetic resonance imaging, dynamic causal modeling, effective connectivity, fronto-thalamic circuit

## Abstract

This study aims to investigate alterations in effective connectivity (EC) within the fronto-thalamic circuit and their associations with motor and cognitive declines in pontine infarction (PI). A total of 33 right PI patients (RPIs), 38 left PI patients (LPIs), and 67 healthy controls (HCs) were recruited. The spectral dynamic causal modeling (spDCM) approach was used for EC analysis within the fronto-thalamic circuit, including the thalamus, caudate, supplementary motor area (SMA), medial prefrontal cortex (mPFC), and anterior cingulate cortex (ACC). The EC differences between different sides of the patients and HCs were assessed, and their correlations with motor and cognitive dysfunctions were analyzed. The LPIs showed increased EC from the mPFC to the R-SMA and decreased EC from the L-thalamus to the ACC, the L-SMA to the R-SMA, the R-caudate to the R-thalamus, and the R-thalamus to the ACC. For RPIs, the EC of the R-caudate to the mPFC, the L-thalamus and L-caudate to the L-SMA, and the L-caudate to the ACC increased obviously, while a lower EC strength was shown from the L-thalamus to the mPFC, the LSMA to the R-caudate, and the R-SMA to the L-thalamus. The EC from the R-caudate to the mPFC was negatively correlated with the MoCA score for RPIs, and the EC from the R-caudate to the R-thalamus was negatively correlated with the FMA score for LPIs. The results demonstrated EC within the fronto-thalamic circuit in PI-related functional impairments and reveal its potential as a novel imaging marker.

## 1. Introduction

With the aging of the population, ischemic stroke has become the first cause of disability and mortality worldwide [1]. As the most common type of posterior circulation infarction, pontine infarction (PI) accounts for 7% of ischemic strokes and is usually caused by the occlusion of the basilar artery or its branches [2,3]. Common symptoms of PI include sensory and motor dysfunction, ataxia, facial paralysis, dysarthria, dysphagia, and cognitive impairment [4,5]. A substantial proportion of patients with pontine infarction suffer from long-term motor or cognitive dysfunction, causing a great burden on the healthcare and social welfare system [6,7]. Understanding the underlying neural mechanisms of behavioral dysfunction is crucial for severity assessment and remote rehabilitation in PI.

In recent years, resting-state functional magnetic resonance imaging (rs-fMRI) has been widely used to investigate the neurobiological mechanisms underlying different conditions. Apart from local ischemic insults, a pontine lesion may also cause damage to the functional area in the cortex far from the pontine region and the anatomical pathways between the cortex and the pontine [8,9]. Rewiring surviving brain circuits may be a crucial compensation for the function deficit caused by stroke. As a complex neural pathway consisting of the medial prefrontal cortex (mPFC), the anterior cingulate cortex (ACC), the thalamus, and other subcortical structures, the fronto-thalamic circuit plays an important role in various cognitive and behavioral functions. The fronto-thalamic circuit also participates in some essential functions, such as attention, decision making, working memory, motor control, and emotional regulation [10]. Some studies have indicated that damage to the fronto-cerebellar-thalamic loop may be the potential cause of cognitive decline in patients with pontine stroke [11]. A study performed by Jiang et al. revealed that patients with pontine infarction showed gray matter volume (GMV) reduction in the anterior insular cortex and cerebellum [12]. Increased GMVs were also observed in the supplementary motor area (SMA). Our previous study also revealed that PI patients showed disturbed structural and functional connectivity; a positive correlation was also observed between structural connectivity and functional connectivity coupling and movement assessment scores [13]. Another study using three-dimensional pseudo-continuous arterial spin labeling (3D-pcASL) and rs-fMRI scans suggested that patients in the left PI showed a significant change in CBF in the right SMA; a functional connectivity change was also found in the prefrontal lobe [6]. Thus, fronto-thalamic circuitry dysfunction might underlie PI pathogenesis, and further investigations are needed to identify the exact mechanisms. 

Different from functional connectivity, which only explores the relationship between different brain areas, effective connectivity (EC) can further investigate the directionality of coupling. Based on the spectral dynamic causal model (spDCM) proposed by Friston et al., the brain’s effective connectivity within different networks can be analyzed and can characterize their causal relationship [14]. However, spDCM has been widely used to explore different pathological conditions, including schizophrenia, depression, and the long-term behavior of chronic stroke survivors [15,16,17]. No studies have been performed to investigate effective connectivity in PI patients or its relevance to motor and cognitive functions. As the fronto-thalamic circuit plays a crucial role in cognitive and motor functions, changes in EC in the fronto-thalamic circuit may be the neurological underpinnings of motor and cognitive deficits in PI patients.

In the present study, we used spDCM analysis to explore the characteristics of brain activity changes in PI patients. The brain structures of the fronto-thalamic circuit in both hemispheres were included, i.e., the bilateral thalamus, bilateral supplementary motor area (SMA), bilateral caudate, medial prefrontal cortex (mPFC), and ACC. The relationship between alterations in functional connectivity and motor and cognitive deficits was also analyzed, hoping to identify the altered pattern in brain activity following pontine infarction and provide novel imaging biomarkers for early prediction of cognitive and motor dysfunction in pontine infarction patients.

## 2. Materials and Methods

### 2.1. Study Design

Pontine stroke patients were recruited from the Department of Neurology, Nanjing First Hospital, between March 2018 and August 2022. The patients underwent examinations in the acute phase after a stroke (<3 days). The neurological assessment included items regarding motor and cognitive functions on the same day after an MRI examination. HCs matched in age, gender, and education level were enrolled via a community health census. In the HC group, they underwent only MRI examination and cognitive function assessment.

### 2.2. Demographic and Clinical Data

This study was approved by the ethics committee of Nanjing Medical University, and all participants provided written informed consent. The inclusion criteria for patients were: (1) first-time ischemic stroke; (2) right-handedness; (3) examination within 3 days of stroke onset; and (4) single lesion involvement. The exclusion criteria were: (1) contraindications for MRI; (2) anamnesis of psychiatric or neurologic disorders; (3) other brain disorders discovered in magnetic resonance images; and (4) a modified Fazekas scale for white matter hyperintensities of >1. Healthy adults were recruited for this study, with the exclusion criteria comprising (1) serious mental illness (e.g., psychosis, bipolar disorder, or obsessive-compulsive disorder) and (2) MRI contraindications. The groups were matched for age, gender, and education. A total of forty LPIs, thirty-seven RPIs, and seventy HCs participated. Two LPIs, four RPIs, and three HCs were excluded due to head motion artifacts. 

The National Institutes of Health Stroke Scale (NIHSS) score was assessed on admission (with greater scores indicating increasing severity). The motor function of stroke patients was also recorded using the Fugl–Meyer Assessment (FMA), including the whole extremity and the upper limb tests. In addition, cognition was assessed using the Montreal Cognitive Assessment (MoCA); if the number of years of education was ≤12, one point was added.

### 2.3. MRI Acquisition

The MR images of all subjects were collected on a 3.0-Tesla scanner (Ingenia, Philips Medical Systems, Eindhoven, The Netherlands) equipped with an eight-channel head coil. During the scan, all subjects were asked to keep still and try not to think about anything with their eyes closed. Foam padding and earplugs were used to minimize the noise effects of involuntary head motion. rs-Fmri data were obtained in the axial plane with a gradient echo-planar imaging (EPI) sequence with the following parameters: repetition time = 2000 ms, echo time = 30 ms, flip angle (FA) = 90°, field of view (FOV) = 240 mm × 240 mm, slice = 36, thickness = 4 mm without interslice gap, matrix size = 64 × 64, and the total time = 8 min and 8 s. Sagittal three-dimensional T1-weighted images (3D-T1WI) were obtained using a brain volume sequence with the following parameters: TR = 8.1 ms, TE = 3.7 ms, slices = 170, thickness = 1 mm, gap = 0 mm, fractional anisotropy (FA) = 8°, acquisition matrix = 256 × 256, FOV = 256 mm × 256 mm), and the total time = 5 min and 29 s. 

### 2.4. Data Analysis

#### 2.4.1. MRI Data Preprocessing

The preprocessing of the rs-fMRI data was carried out using the GRETNA software (V.2.0.0, Beijing, China), which was based on the MATLAB 2013b platform. The first step involved converting the DICOM files to NIFTI format and removing the initial 10 volumes from each time series. The subsequent step involved correcting the remaining 220 volumes for acquisition time delay between slices (slice timing) and head motion (realignment). Any data that exhibited translational or rotational head motion greater than 2.0 mm or 2.0° throughout the scan were excluded. The rest of the dataset was then spatially normalized using the Montreal Neurological Institute template, with a resampling voxel size of 3 mm × 3 mm × 3 mm. The normalized fMRI data were then smoothed using a 6 mm full width at half-maximum Gaussian kernel. 

#### 2.4.2. Spectral Dynamic Causal Modeling Analysis

The first stage of analysis involved applying a general linear model to the time series, including the white matter signal, six rigid body motion parameters, and the cerebrospinal fluid signal as nuisance covariates for the first-level analysis. The second stage involved defining regions of interest (ROIs) to construct the DCM model using the preprocessed fMRI data. Based on the previous literature, we defined ROIs as having a radius of 6 mm, and their MNI coordinates were as follows: thalamus (±12, −19, 8), caudate (±16, 0, 18), mPFC (−1, 55, −3), ACC (−1, 12, 38), and SMA (±8, 11, −67). The first eigenvariate of the time series was obtained from these ROIs. To eliminate spurious influence caused by white matter and cerebrospinal fluid, a GM mask was used in the time series with a threshold of 0.5. Then, dynamic causal modeling was performed using the selected ROIs. For each subject, bidirectional connections between any two ROIs were analyzed, and the parameters of the fully connected model were obtained. The Parametric Empirical Bayes (PEB) framework was used to perform the model estimation. A single spDCM model was estimated jointly and followed by Bayesian model reduction. Finally, the effective connectivity value was obtained from the estimation results.

#### 2.4.3. Statistical Analyses

It is worth noting that in previous studies related to ischemic stroke, flipping of the hemisphere was usually adopted before processing the data. However, the hemispheric asymmetry of the brain has been well documented. Therefore, we analyzed the right lesion and left lesion separately instead of flipping the lesion side.

The statistical analysis of the demographic and clinical data was conducted using the SPSS 26.0 software package (SPSS, Inc., Chicago, IL, USA). One-way analysis of variance (ANOVA) among the three groups was used to detect differences in age and years of education, and a chi-square test was used to compare gender differences between the groups, with significance levels set at *p* < 0.05. For spDCM analysis, independent two-sample *t* tests were performed to compare mean connectivity strength differences between patients and healthy controls, with significance levels set at *p* < 0.05.

The ROC curve was used to analyze the differences in mean connectivity strength differences between the patients and HCs. The closer the area under the curve (AUC) was to 1, the more favorable the diagnosis. In addition, Pearson’s correlation was utilized to assess the correlation between the MoCA score, FMA score, and effective connectivity strength. The statistical threshold was set at *p* < 0.05.

## 3. Results

### 3.1. Demographic and Clinical Data

**Figure 1:**
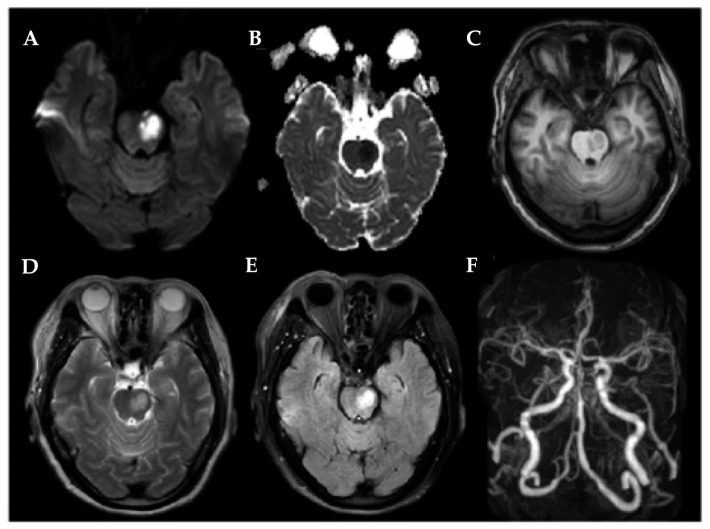
A 59-year-old female patient was referred for a left pontine infarction case. (**A**) High signal on DWI axial images; (**B**) low signal on ADC; (**C**) low signal on T1-weighted section; (**D**) high signal on T2-weighted section; (**E**) high signal on FLAIR; (**F**) the MRA 3D-TOF confirmed basilar artery stenosis.

**Figure 2:**
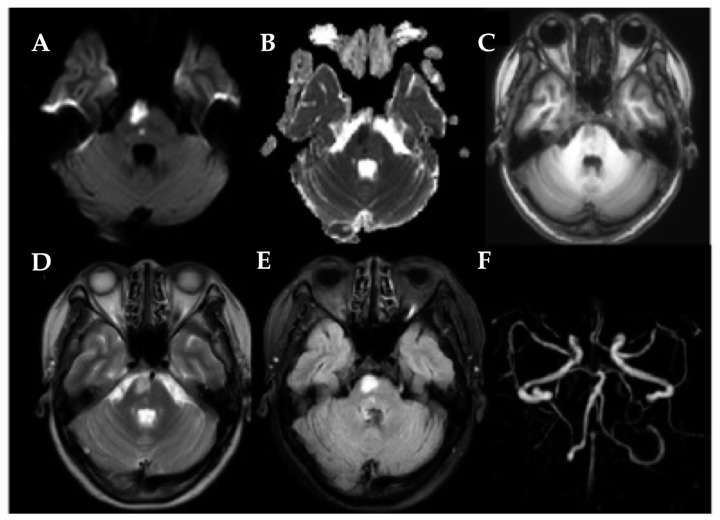
A 55-year-old female patient was referred for a right pontine infarction case. (**A**) High signal on DWI axial images; (**B**) low signal on ADC; (**C**) low signal on T1-weighted section; (**D**) high signal on T2-weighted section; (**E**) high signal on FLAIR; (**F**) no evidence of vascular occlusion on the MRA 3D-TOF.

Finally, 38 LPIs (Figure 1), 33 RPIs (Figure 2), and 67 HCs were included. Detailed demographic, clinical, and behavioral data are described in Table 1. There were no significant differences in age, gender, or years of education between the unilateral PI and the HCs (all *p* > 0.05, Table 1). Compared with the HC, PI patients performed worse in the MoCA.

### 3.2. Effective Connectivity Analysis

**Figure 3:**
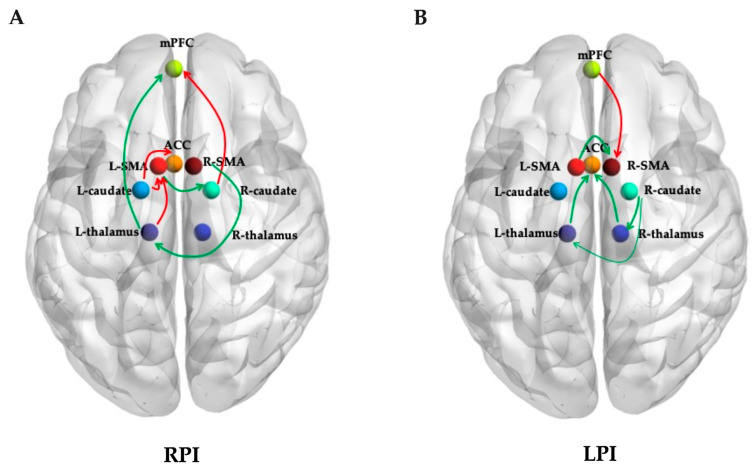
Results of resting state effective connectivity analysis: (**A**) effective connectivity with significant difference between HCs and RPIs; (**B**) effective connectivity with significant difference between HCs and LPIs. Red arrow, EC significantly higher; green arrow, EC significantly lower; RPI, right pontine stroke; LPI, left pontine stroke; EC, effective connectivity; ACC, anterior cingulate cortex; SMA, supplementary motor area; mPFC, medial prefrontal cortex; L, left; R, right.

**Figure 4:**
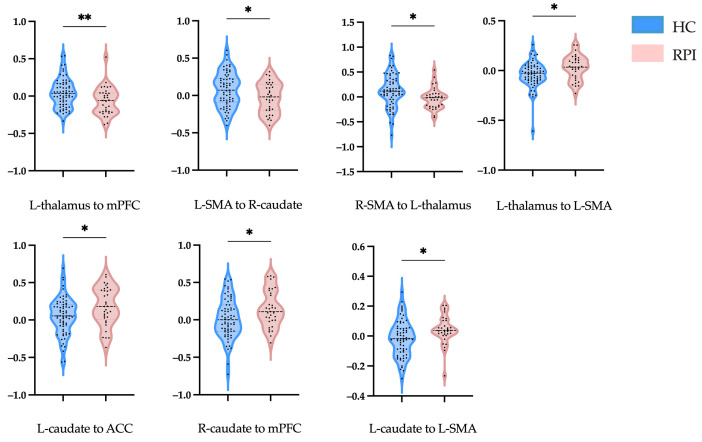
Effective connectivity strengths in HCs and RPIs. Violin illustration shows the connection with a significant difference between the two groups. The abscissa axes represent the different groups, and the ordinate axes represent the effective connectivity strengths. HCs, healthy controls; RPI, right pontine infarction; L, left; R, right; mPFC, medial prefrontal cortex; SMA, supplementary motor area; ACC, anterior cingulate cortex. * Indicates significance at the 0.05 significance level; ** indicates significance at the 0.01 significance level.

To investigate the differences between groups, an independent sample t test was performed to compare the mean connectivity strength. For RPIs, the effective connectivity increased from the R-caudate to the mPFC, from the L-thalamus and L-caudate to the L-SMA, and from the L-caudate to the ACC, while a lower effective connectivity strength was shown from the L-thalamus to the mPFC, from the L-SMA to the R-caudate, and from the R-SMA to the L-thalamus (Figure 3A and Figure 4, Table 2).

**Figure 5:**
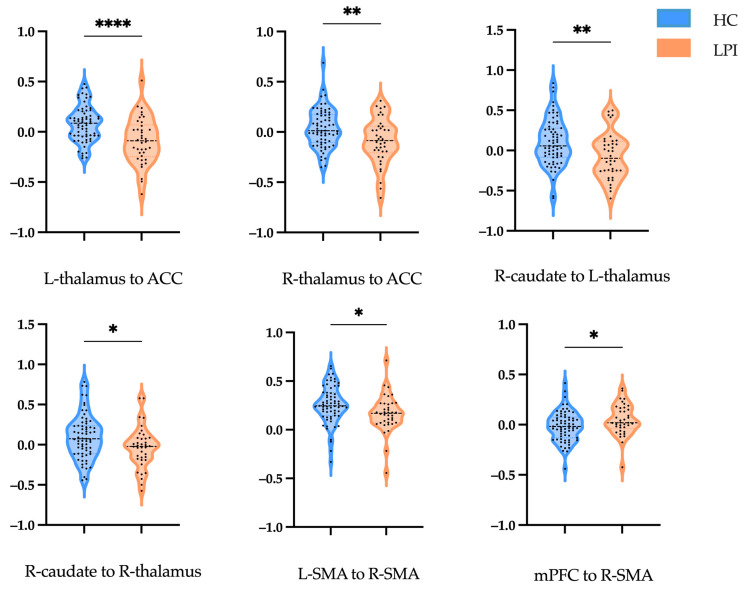
Effective connectivity strengths in HCs and LPIs. Violin illustration shows the connection with a significant difference between the two groups. The abscissa axes represent the different groups, and the ordinate axes represent the effective connectivity strengths. HC, healthy control; LPI, left pontine infarction; L, left; R, right; ACC, anterior cingulate cortex; SMA, supplementary motor area; mPFC, medial prefrontal cortex. * Indicates significance at the 0.05 significance level; ** indicates significance at the 0.01 significance level; **** indicates significance at the 0.001 significance level.

For the LPIs, the effective connectivity increased from the mPFC to the R-SMA, while a lower effective connectivity strength was shown from the left and right thalamus to the ACC, from the R-caudate to the left and right thalamus, and from the L-SMA to the R-SMA (Figure 3B and Figure 5, Table 3).

### 3.3. Receiver Operating Characteristic Curve

**Figure 6:**
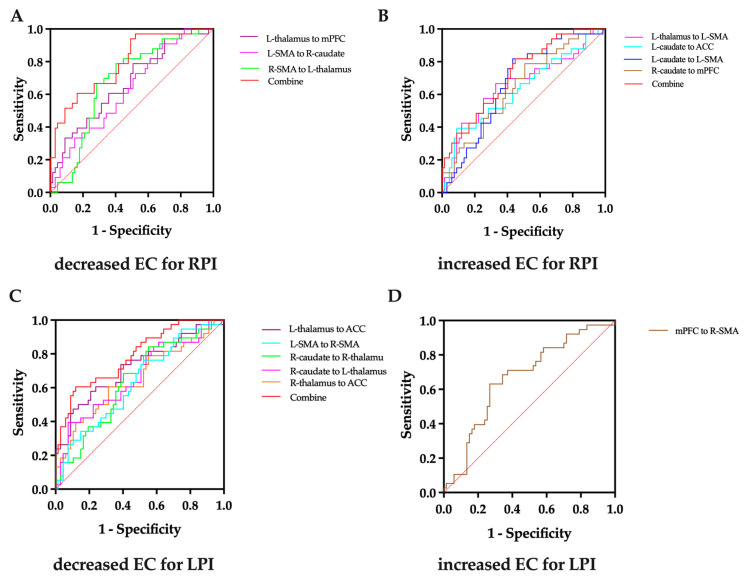
ROC curve analysis of effective connectivity strength differences. (**A**) Decreased effective connectivity strength differences for RPIs. (**B**) Increased effective connectivity strength differences for RPIs. (**C**) Decreased effective connectivity strength differences for LPIs. (**D**) Decreased effective connectivity strength differences for LPIs. EC; effective connectivity; LPI, left pontine stroke; RPI, right pontine stroke; L, left; R, right; mPFC, medial prefrontal cortex; SMA, supplementary motor area; ACC, anterior cingulate cortex. The red line represents the diagonal of the ROC curve.

The effective connectivity was further used in an ROC to evaluate the ability to distinguish the patients from the HC group. The results of the AUC analysis of the effective connectivity for RPIs were as follows: 0.667 for the L-thalamus to the mPFC, 0.629 for the L-SMA to the R-caudate, 0.678 for the R-SMA to the L-thalamus, 0.642 for the R-SMA to the L-thalamus, 0.789 for a combination of these decreased effective connectivity changes together, 0.657 for the L-thalamus to the L-SMA, 0.633 for the L-caudate to the ACC, 0.645 for the R-caudate to the mPFC, 0.669 for the L-caudate to the L-SMA, and 0.721 for a combination of these increased effective connectivity changes together (Figure 6A,B). The results of the AUC analysis of the effective connectivity for LPIs were 0.719 for the L-thalamus to the ACC, 0.650 for the R-thalamus to the ACC, 0.663 for the R-caudate to the L-thalamus, 0.639 for the R-caudate to the R-thalamus, 0.630 for the L-SMA to the R-SMA, 0.788 for a combination of these decreased effective connectivity changes together, and 0.667 for the mPFC to the R-SMA (Figure 6C,D).

### 3.4. Correlation Analyses

**Figure 7:**
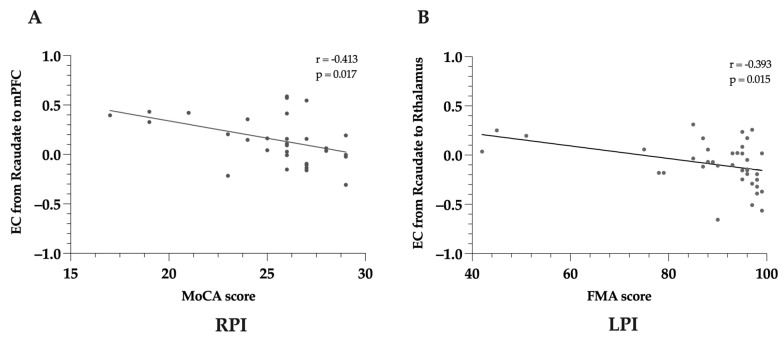
Pearson’s correlation between effective connectivity strength differences and behavioral characteristics of patients. (**A**) Correlation between effective connectivity strengths and MoCA score for RPIs; (**B**) effective connectivity strengths and FMA scores for LPIs. FMA, Fugl–Meyer assessment; LPI, left pontine stroke; R, right; RPI, right pontine stroke; EC, effective connectivity; mPFC, medial prefrontal cortex; MoCA, Montreal Cognitive Assessment.

The correlations between altered EC and clinical assessments are shown in Figure 7. Our study revealed that a significant negative correlation between the EC from the R-caudate to the mPFC and the MoCA score in RPIs. The EC from the R-caudate to the R-thalamus was also markedly associated with motor function in the LPIs. 

## 4. Discussion

Using the spDCM method, we explored the EC within the fronto-thalamic circuit and its correlations with motor and cognitive function in PI patients. As hypothesized, patients with pontine infarction showed significant EC changes in the fronto-thalamic circuit, and different reconstruction modes were seen in the right and left PI individuals. The effective connectivity from the R-caudate to the mPFC was significantly negatively correlated with the MoCA score for the RPIs, while the effective connectivity from the R-caudate to the R-thalamus was significantly negatively correlated with the FMA score for the LPIs. These findings suggested that unilateral PI can cause damage to direct or indirect functional connectivity between supratentorial regions in both the ipsilateral and contralateral hemispheres. Different remodeling and compensatory mechanisms may exist in right and left pontine lesions, which might be the underlying neural and pathophysiological cause of different clinical manifestations and long-term outcomes. Novel imaging biomarkers for the early prediction of cognitive and motor dysfunction in PI patients may be identified, and possible targets for therapeutic intervention may be found in the future studies.

Apart from local ischemic injury, patients with PI also presented with diverse manifestations related to the functional impairment of the supratentorial cortical areas, which are remote from the pontine region [5]. A study performed by Wang et al. revealed that the ratio between whole-brain CBF and functional connectivity strength (FCS) was obviously interrupted in PI patients [18]. As one of the organs with the highest energy expenditure, the brain maintains its normal function by keeping an optimum balance between blood supply and neural activity. The CBF in different supratentorial regions was decreased in right and left PI patients, suggesting that decreased neurovascular coupling may be the neural basis underlying behavioral dysfunction after PI. Using static and dynamic metrics, Ren et al. observed that patients with PI displayed abnormal brain activity in both motor and cognitive systems [19]. In line with our present study, significant differences in brain activity changes were seen in right and left PI patients. Thus, a lesion-side effect may exist in patients with subtentorial infarction. 

The SMA plays an important role in facilitating the initiation and coordination of self-initiated movements. Previous studies have revealed that the SMA has a rich interconnectivity with other cortical regions and subcortical structures. Using healthy individuals and experimental rats, Çavdar S et al. explored the connections between the SMA and brainstem structures, and both efferent and afferent projections were seen between the SMA and pontine [20]. Another previous study also found that the SMA participates in the formation of the corticospinal tract (CST), making up about 10% of corticospinal cells [21]. During different motor activities, activations of the SMA and the primary motor (M1) were both observed in a single-neuron recording study [22]. In our present study, we confirmed that the connections of the L-SMA to the R-caudate in the RPI group were significantly lower than those in HCs, but the connections of the L-thalamus to the L-SMA and the L-caudate to the L-SMA were obviously increased compared to those in the controls. Therefore, for patients with right pontine infarction, the effective connectivity between ipsilateral SMA and other brain areas were reduced significantly. The connections between contralateral SMA and other regions increased markedly, which might be a compensatory mechanism to ameliorate the motor functional impairment caused by contralateral pontine infarction. For patients with LPI, the connection of the L-SMA to the R-SMA was decreased, while that from the mPFC to the R-SMA was increased obviously compared with the controls. Different brain functional connectivity changes caused by location-specific infarction may be the neural mechanisms underlying distinct clinical manifestations and functional recovery after PI.

Another important finding of our present study was that the EC from the R-caudate to the R-thalamus and L-thalamus decreased significantly in patients with LPI. Located in the diencephalon, the thalamus is a nuclear complex that relays nearly all incoming information to the cortex. Several cortical regions receive afferent fibers from a single thalamic nucleus and send back efferent fibers to distinct thalamic nuclei [23]. Connecting to the cerebral cortex, hippocampus, and brainstem, the thalamus plays a fundamental role in perception, motor control, and consciousness. Through altering the transmission of information throughout basal ganglia circuits, the striatum is a critical neural substrate for motor control and procedural memory [24]. Serving as critical nodes in the cortico-striato-thalamo-cortical loop, the functions of the thalamus and caudate and their connections with other regions have been widely studied in previous research [25,26]. The connections between the dorsomedial nucleus of the thalamus, rostral and dorsal caudate nuclei, and the dorsolateral prefrontal cortex are mainly involved in cognitive function and form the “cognitive” loop [27]. Additionally, the thalamus is also connected to the brainstem and cortex via the cortico-ponto-cerebello-thalamo-cortical circuitry, which also contains cognitive and motor loops. Though it was previously considered to be parallel to the cortico-striato-thalamo-cortical circuitry, considerable overlaps were found between the cortico-ponto-cerebello-thalamo-cortical and cortico-striato-thalamo-cortical pathways at the thalamic level in a recent study [28]. Our present study revealed that the connections from the R-caudate to the R-thalamus and L-thalamus in LPIs decreased significantly compared with the controls. Thus, in addition to the direct damage to the loop caused by pontine infarction, an indirect injury to the loop may also be present. The remarkable decline in the loop maybe an important neural mechanism underlying motor loss and cognitive decline, especially for those with left pontine infarction. Abnormal impairment in thalamic circuits has been recognized as the basis for many neurodevelopmental disorders, including schizophrenia, obsessive compulsive disorder, and autism spectrum disorder [13,29]. Abnormal development of the thalamic components of the cortico-striato-thalamo-cortical circuit may also play a critical role in many neurodevelopmental disorders [26]. 

The ACC and mPFC are important components of the default mode network (DMN), which is involved in introspection, self-referential mental activity, and social cognition. These regions are closely interconnected and play major roles in emotional and cognitive processing [30]. The results of our present study revealed that the connections of EC from the L-thalamus and R-thalamus to the ACC were significantly decreased in the LPI group, while the connection of the L-thalamus to the mPFC in the RPI group was obviously lower than those in the controls. Located in the frontal part of the brain, the ACC is involved in a wide range of functions, including error detection, conflict monitoring, and regulation of affect and motivation. The ACC receives inputs from distinct brain regions, including the amygdala, hippocampus, and somatosensory cortex, and projects to various areas such as the mPFC, striatum, and motor cortex. The mPFC is also located in the frontal lobe and is involved in many neurological functions, including social cognition, self-referential processing, and introspection. It receives input from areas such as the temporal and parietal lobes and projects to the subgenual ACC and striatum. The ACC and mPFC are highly interconnected through a network of brain regions and have strong functional connectivity during rest and cognitive tasks, suggesting a close relationship between these brain regions [31,32]. Overall, the ACC and mPFC play crucial roles in emotional and cognitive processing through their network connections with other brain regions. Understanding their function and connectivity is important for understanding the neural mechanisms underlying cognitive and emotional function impairment.

## 5. Limitations

Several limitations exist in our present study. Firstly, though the thalamus consists of several different nuclei, including the ventral anterior and ventral lateral nuclei, the thalamus was set as one ROI to determine its relationships with other regions. More detailed studies should be conducted in the future to elucidate the specific effect of different nucleus. Secondly, the sample size of our cohort of pontine infarction cohort was relatively small; thus, future studies with more patients are needed to further confirm these preliminary results. Lastly, two LPIs, four RPIs, and three HCs were excluded from the study due to head motion artifacts during the MRI scan. This exclusion criterion may have introduced a selection bias in the sample, potentially limiting the generalizability of the findings. Future studies should consider alternative methods to minimize head motion during scanning.

## 6. Conclusions

The present study revealed that patients with pontine infarction experienced different EC changes in the fronto-thalamic circuit, and different reconstruction modes were seen in the right and left PI individuals. The effective connectivity from the R-caudate to the mPFC was significantly negatively correlated with the MoCA score for RPIs, while the effective connectivity from the R-caudate to the R-thalamus was significantly negatively correlated with the FMA score for LPIs. We demonstrate herein that unilateral PI can cause damage to direct or indirect functional connectivity between supratentorial regions. The results support and reinforce the role of abnormal effective connectivity within the fronto-thalamic circuit in the pathophysiology of pontine-stroke-related motor and cognitive impairment and reveal its potential as a novel imaging marker. Future studies with long-term follow-up periods will provide more insights into the pathophysiology underlying neurological recovery and may be further used to develop novel treatment strategies.

## Figures and Tables

**Table 1:** Demographic characteristics and neuropsychological scores of pontine stroke patients and healthy controls.

	RPI (*n* = 33)	LPI (*n* = 38)	HCs (*n* = 67)	*p*-Value
Age (years)	65.76 ± 8.98	64.53 ± 10.64	61.84 ± 6.60	0.067
Gender (male/female)	21/12	26/12	45/22	0.906
Education (years)	10.06 ± 1.82	10.95 ± 2.21	10.97 ± 1.87	0.319
NIHSS score	3.12 ± 2.26	3.50 ± 2.29	-	0.486
FMA score	85.68 ± 14.62	88.79 ± 14.09	-	0.349
MoCA score	25.45 ± 2.91	25.47 ± 3.59	26.09 ± 2.672	0.016 *

RPI, right pontine infarction; LPI, left pontine infarction; HCs, healthy controls; FMA, Fugl–Meyer assessment. * Statistical significance.

**Table 2:** Group differences in mean effective connectivity strength for regions of interest in RPIs.

Connection	Connectivity Strength (Mean ± SD)		*p*-Value	t
	HCs	RPI		
L-thalamus to mPFC	0.424 ± 0.192	−0.066 ± 0.185	0.008	2.737
L-SMA to R-caudate	0.074 ± 0.231	−0.032 ± 0.202	0.027	2.246
R-SMA to L-thalamus	0.126 ± 0.326	−0.028 ± 0.207	0.005	2.882
L-thalamus to L-SMA	−0.039 ± 0.128	−0.295 ± 0.121	0.010	−2.634
L-caudate to ACC	0.053 ± 0.249	0.177 ± 0.259	0.023	−2.310
L-caudate to LSMA	−0.016 ± 0.119	0.042 ± 0.094	0.016	−2.441
R-caudate to mPFC	0.014 ± 0.264	0.148 ± 0.246	0.016	−2.454

HCs, healthy controls; RPI, right pontine infarction; mPFC, medial prefrontal cortex; SMA, supplementary motor area; ACC, anterior cingulate cortex.

**Table 3:** Group differences in mean effective connectivity strength for regions of interest in LPIs.

Connection	Connectivity Strength (Mean ± SD)		*p*-Value	t
	HCs	LPI		
L-thalamus to ACC	−0.812 ± 0.179	−0.088 ± 0.229	<0.001	4.205
R-thalamus to ACC	0.036 ± 0.190	−0.091 ± 0.229	0.003	3.035
R-caudate to L-thalamus	0.103 ± 0.291	−0.066 ± 0.286	0.005	2.868
R-caudate to R-thalamus	0.098 ± 0.277	−0.034± 0.254	0.015	2.422
L-SMA to R-SMA	0.260 ± 0.192	0.166 ± 0.188	0.017	2.425
mPFC to R-SMA	−0.015 ± 0.149	0.049 ± 0.152	0.038	−2.101

HC, healthy control; LPI, left pontine infarction; ACC, anterior cingulate cortex; SMA, supplementary motor area; mPFC, medial prefrontal cortex.

## Data Availability

Data supporting the results of this study are available from the corresponding author upon reasonable request. The data are not publicly available due to privacy concerns.

## Abbreviations

ACC	Anterior cingulate cortex	mPFC	Medial prefrontal cortex
EC	Effective connectivity	NIHSS	National Institutes of Health Stroke Scale
FMA	Fugl–Meyer Assessment	PI	Pontine infarction
GMV	Gray matter volume	RPI	Right pontine infarction
HC	Healthy control	rs-fMRI	Resting-state functional magnetic resonance imaging
LPI	Left pontine infarction	spDCM	Spectral dynamic causal modeling
MoCA	Montreal Cognitive Assessment	SMA	Supplementary motor area
